# A computerized tablet with visual feedback of hand position for functional magnetic resonance imaging

**DOI:** 10.3389/fnhum.2015.00150

**Published:** 2015-03-25

**Authors:** Mahta Karimpoor, Fred Tam, Stephen C. Strother, Corinne E. Fischer, Tom A. Schweizer, Simon J. Graham

**Affiliations:** ^1^Graham Laboratory, Physical Sciences Platform, Sunnybrook Research Institute, Sunnybrook Health Sciences CentreToronto, ON, Canada; ^2^Department of Medical Biophysics, University of Toronto Faculty of MedicineToronto, ON, Canada; ^3^Strother Laboratory, Rotman Research InstituteBaycrest, Toronto, ON, Canada; ^4^Heart and Stroke Foundation Canadian Partnership for Stroke RecoveryCanada; ^5^Geriatric Psychiatry, Psychiatry Department, St. Michael’s HospitalToronto, ON, Canada; ^6^Department of Psychiatry, University of Toronto Faculty of MedicineToronto, ON, Canada; ^7^Keenan Research Centre for Biomedical Science of St. Michael’s HospitalToronto, ON, Canada

**Keywords:** computerized tablet, ecological validity, fMRI, handwriting, human factors, neuropsychological tests, proprioception, visual feedback

## Abstract

Neuropsychological tests behavioral tasks that very commonly involve handwriting and drawing are widely used in the clinic to detect abnormal brain function. Functional magnetic resonance imaging (fMRI) may be useful in increasing the specificity of such tests. However, performing complex pen-and-paper tests during fMRI involves engineering challenges. Previously, we developed an fMRI-compatible, computerized tablet system to address this issue. However, the tablet did not include visual feedback of hand position (VFHP), a human factors component that may be important for fMRI of certain patient populations. A real-time system was thus developed to provide VFHP and integrated with the tablet in an augmented reality display. The effectiveness of the system was initially tested in young healthy adults who performed various handwriting tasks in front of a computer display with and without VFHP. Pilot fMRI of writing tasks were performed by two representative individuals with and without VFHP. Quantitative analysis of the behavioral results indicated improved writing performance with VFHP. The pilot fMRI results suggest that writing with VFHP requires less neural resources compared to the without VFHP condition, to maintain similar behavior. Thus, the tablet system with VFHP is recommended for future fMRI studies involving patients with impaired brain function and where ecologically valid behavior is important.

## Introduction

Neuropsychological (NP) tests are an important tool kit for clinical psychologists, behavioral neurologists, psychiatrists and related medical professionals. Traditionally conducted mostly in pencil and paper format, but now increasingly with computers (Wild et al., 2008), NP tests are standardized behavioral tasks (including scoring mechanisms) that have been carefully designed specifically to measure mental processes that are thought to be linked with one or more specific brain structures. In practice, NP tests probe various aspects of human cognition, ability, or skill, with the intent to detect and characterize abnormal brain function, and to distinguish abnormal from normal brain function (Hebben and Milberg, 2009). The behavioral abnormalities measured by NP tests may assist in arriving at a diagnosis (e.g., stroke, brain tumor, Alzheimer’s Disease, traumatic brain injury, and depression) and may assist clinicians to identify a treatment target and a treatment plan.

However, it is well known that the relationship between NP test scores and impaired brain function is complicated. Because behavioral performance is the outcome of regionally distributed brain activity (Lezak et al., 2004; Strauss et al., 2006), the specificity of NP tests is negatively affected by the fact that a poor test score may arise due to damage to one or more brain regions, or their interconnections. Aside from the standard practice of administering NP tests in batteries to provide convergent validity of findings, another way that is emerging to improve the specificity of NP tests involves simultaneous measurement of behavior and brain activity (Lezak et al., 2004; Hebben and Milberg, 2009). fMRI is an important tool for such an approach (Lezak et al., 2004; Wild et al., 2008; Hebben and Milberg, 2009). The fMRI method is widely recognized as a safe, non-invasive method to probe neuronal activity indirectly through the associated localized changes in blood oxygenation, flow, and volume (Ogawa et al., 1990, 1992).

Since its discovery over two decades ago, fMRI has become ubiquitous in neuroscience research to study brain/behavior relationships. However, the unique challenges of the fMRI examination (e.g., strict limits on electromagnetic interference, avoidance of ferromagnetic components that may experience strong attractive forces, the need to conduct behavioral testing with the patient lying supine within the confines of the magnet bore) have been a barrier to extensive development of peripheral devices for sensory stimulus presentation and behavioral response recording. The vast majority of fMRI studies continue to use button boxes to record finger presses as the only mode of behavioral response (e.g., to indicate “yes” or “no”, or to record reaction times (Gould et al., 2003; Diciotti et al., 2010; de Rover et al., 2011)). Such responses are insufficient if fMRI is to be used to measure brain activity that accurately reflects NP test performance, especially for those tests that involve recording patient responses by writing and drawing behavior.

Our group previously designed and validated a computerized fMRI-compatible tablet system to address this issue (Tam et al., 2011). Rather than attempting to cope with the substantial engineering challenges that are introduced by a user operating an integrated, touch-sensitive video display during fMRI, a simpler, novel approach was adopted that separated the two required pathways for sensory stimulus presentation. The prototype consisted of an opaque touch-sensitive screen that could be operated by finger or using a stylus, with the user viewing visual stimuli and their subsequent tablet interactions with an LCD projector and rear-projection screen combination, through an angled mirror. The tablet system was controlled by and connected to a computer located at the MRI console, providing electronic communication and power requirements through filtered and shielded cables that passed through the penetration panel of the radiofrequency (RF) shield enclosing the magnet room. To date, the prototype has been used in several fMRI studies involving the Trail Making Test (Churchill et al., 2012), a widely used NP test of cognitive function (Halstead, 1947; Stuss et al., 2001), as well as studies of bimanual co-ordination (Callaert et al., 2011) and creative processes (Ellamil et al., 2012), with cohorts of healthy adults.

These initial studies, while successful, have motivated the investigation of a potentially important human factors issue involving the prototype tablet system. While lying supine in the confined magnet bore, users cannot view their hand directly while interacting with the prototype tablet and must rely predominantly on proprioception (sense of body position in space as determined by receptors in muscles and tendons). Unfortunately, proprioception often provides less accurate spatial information for guiding movement than is provided by vision (Welch and Warren, 1986; Gordon et al., 1994; Graziano, 1999). Although the precise weighting of vision and proprioception may vary with the direction of movement (Redding and Wallace, 1996; van Beers et al., 1999, 2002; Haggard et al., 2000), visual feedback plays the primary role for supporting lateral movements typically associated with performing writing and drawing movements on a tablet. As demonstrated in motor tasks conducted with and without sensory feedback from the visual system, viewing the hand and arm improves tactile spatial resolution and acuity (Gordon et al., 1994; Graziano and Gross, 1998; Graziano, 1999; Taylor-Clarke et al., 2002, 2004). These observations exemplify the familiar experience that most people have of fumbling for objects in the dark.

Furthermore, the importance of vision to assist in making appropriate movements is likely to be even greater for patients with impaired brain function, such as those with proprioceptive, motor or cognitive deficits. For example, studies of patients with AD (Ghilardi et al., 1999, 2000; Slavin et al., 1999) have shown that slower motion, increased reaction time, and impaired movement planning occur when visual sensory feedback is absent. Similar effects have been observed in stroke patients with lesions in particular regions of visual-sensorimotor cortex, that cause subsequent loss of tactile sensation in the absence of visual feedback when performing touching movements with the hand (Halligan et al., 1996).

Thus, VFHP is highly desirable when users perform complex hand movements such as writing and drawing with the fMRI-compatible tablet. The purpose of the present work is to augment our fMRI-compatible tablet prototype to integrate VFHP using a video camera and augmented reality display, and to assess the influence of VFHP on behavior. In this initial work, behavioral testing is limited to young healthy adults. However, our long term objective is to assess impaired brain/behavior relationships in patients with AD and Mild Cognitive Impairment (MCI) by conducting tablet-based fMRI of a sensitive NP test that involves writing kinematics (Werner et al., 2006). Consequently, the present investigations focus on writing performance. In addition, two fMRI cases have been undertaken involving young healthy adults to demonstrate the impact of VFHP on tablet performance and brain activity. Overall, the experiments were designed to test the overarching hypothesis that using the tablet with VFHP to perform writing improves tablet performance and reduces brain activity in healthy young adults performing writing tasks.

## Methods

### Revised Tablet Design

**Figure 1:**
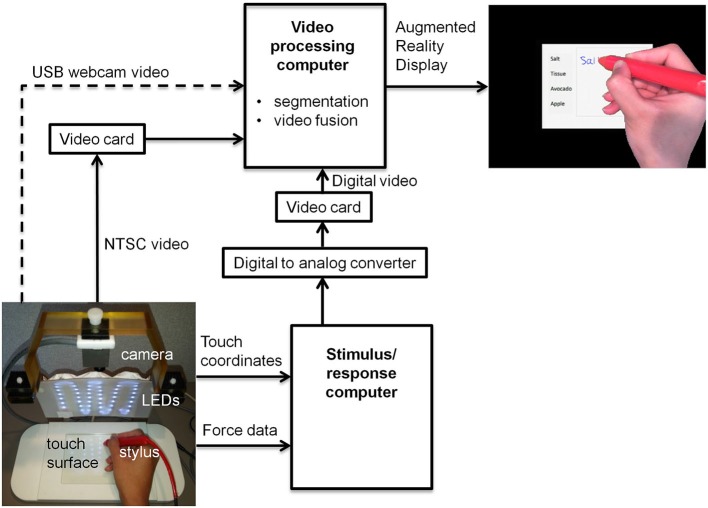
**Block diagram of tablet system design including VFHP**. USB = universal serial bus; LED = light emitting diode; NTSC = National Television System Committee.

Figure 1 shows the block diagram for a proof-of-concept revised fMRI-compatible tablet system that was implemented in the laboratory. At its core, the system included the same resistive touch-sensitive surface used previously for converting localized contact force to position coordinate values, and for locating these values on a computer display (Tam et al., 2011). The area of the touch-sensitive surface was 13 cm × 10 cm. Touch was performed using a stylus, which included a sensor at the tip to measure contact force (FSR 400, 30-49649, Interlink Electronics, Camarillo, CA). The sensor included DC offset and gain adjustments, which in preliminary testing were set to provide a contact sensitivity that was similar to the experience of writing with pen and paper. Touches were recorded with a spatial resolution of 0.13 mm and a report rate of 180 Hz, in communication with a personal computer (Asus N56V, Intel(R) Core™ i7-3610QM CPU @ 2.30 GHz, 8 GB RAM, 64-bit operating system, Windows 7). This “stimulus/response computer” was equipped with a custom program written using E-prime Software (version 2; Psychology Software Tools, Inc., Sharpsburg, PA) that received and interpreted the touch position information, and provided task-related feedback in the form of digital video signals communicated through a Video Graphics Array (VGA) interface. The feedback consisted of tablet responses (e.g., writing and drawing) superimposed on visual stimuli in the form of computer graphics delivered as part of behavioral testing.

To enhance task-related feedback from the tablet, the tablet system was augmented to integrate video recordings of hand/stylus interactions. Provision was made to receive inputs for different video cameras, to permit preliminary behavioral testing at a desktop outside an MRI system, as well as inside an MRI system during fMRI data collection. Experiments outside the MRI system were undertaken using a commercially available universal serial bus (USB) web-camera (LifeCam Studio, Microsoft Inc., Redmond, WA) with a color CMOS sensor at 1920 × 1080 resolution and 30 Hz frame rate (dashed arrow, Figure 1). Experiments undertaken during fMRI used an MRI-compatible video camera with a color CMOS sensor (12M-i, MRC Instruments Gmbh, Germany) mounted vertically above the tablet on a customized frame, 20.5 cm distant from the tablet surface. In this case, NTSC video signals were acquired at 640 × 480 resolution at 30 Hz. The latter configuration included an array of MRI-compatible light emitting diodes (LEDs) to illuminate the video field. In both cases, the video camera field of view was chosen to include the entire touch panel surface and a portion of its surrounding supporting frame.

A second “video processing computer” (Intel Desktop Board DQ97TM, i5 Core™ CPU, 760 @ 2.8 GHz, Windows 7 Professional) acted on video signals coming from a given video camera, and those from the stimulus/response computer. Signals from the web camera were directly accessible through a computer USB port, whereas the analog video signals from the MRI-compatible camera were converted to digital video using a cost-effective video capture card (Impact-VCB-e Hauppauge Win TV 885, Hauppauge Computer Works Inc., Hauppauge, NY). Rather than implementing digital-digital conversion of the VGA signals from the stimulus/response computer, for simplicity these signals were first processed by a digital-to-analog video converter (PCTOTV KW-SA235 KWorld Computer Inc., Brea, CA) followed by use of a second video capture card, identical to the one mentioned above.

Using custom programs written in MATLAB (The Mathworks Inc., Natick, MA) the video-processing computer performed two main manipulations of the incoming data. First, a real-time segmentation procedure was used to isolate the hand and stylus from each camera video frame, using a simple skin color detection algorithm performed in Red-Green-Blue (RGB) space (Kovac et al., 2003). The algorithm exploited the special color distribution of skin (Kovac et al., 2003) compared to the distribution of tablet colors in the background. The skin color segmentation rule based on uniform daylight illumination (Kovac et al., 2003) was utilized as the starting point to cluster skin-colored pixels, with slight adjustment of the R and G threshold values to increase skin detection efficiency in the lab and MRI environments. Each pixel in the RGB image that fell within values defined below was clustered as skin:

(1) $$\begin{array}{l} R>55\&G>20\&B>20\&\\ \max\left\{R,G,B\right\}-\min\left\{R,G,B\right\}>15\&\\ \left|R-G\right|>15\&R>G\&R>B \end{array}$$

In addition, the color properties of the stylus were also adjusted to fall within the RGB distribution of skin color, using a red plastic cover. This ensured that both stylus and hand were segmented appropriately, providing a binary mask image ***M*** of ones and zeros for each camera video frame.

Second, the camera and stimulus/response video signals were superimposed according to the following operation:

(2) $$S_{i}=(C_{i}\times M_{i})+(T_{i}\times \sim M_{i}),$$

Where ***C***_i_ is the camera video frame, ***T***_i_ is the stimulus/response video frame, ***S***_i_ is the superimposed video frame, the multiplier denotes element by element matrix multiplication and the ~symbol denotes the logical “not” operator. The resulting superimposed video signals were then displayed in real-time to the user as an interactive augmented reality environment. A computer display was used for desktop testing (LG Flatron W2442PA-BF Widescreen LCD, 1920 × 1080 resolution, 75 Hz vertical refresh rate, 83 KHz horizontal refresh rate) outside the MRI system, or with an MRI-compatible LCD projector (SV-6011, 1024 × 768 resolution, refresh rate 60 Hz, Avotec Inc., Stuart, FL) and rear-projection screen during fMRI experiments.

Notably, both computers were run asynchronously in this proof-of-concept implementation. Because the video camera data streamed at the lowest frame rate, the most recently acquired VGA data from the stimulus/response computer were paired with each video camera frame to create the augmented reality video. The maximum time lag between each *C_i_* and *T_i_* pair was approximately 16.7 ms. All video processing was achieved within the 30 Hz frame rate of the video camera, ensuring a maximum time lag of 33 ms between tablet interactions and subsequent depiction in the augmented reality display. This is a smaller lag than is required for signals to travel from proprioceptive or visual receptors to primary sensory cortex (approximately 50 ms) (Greenberg and Ducker, 1982). Thus, any potential conflict arising from time delay between visual and proprioceptive sensory signals was considered negligible from the hardware implementation standpoint.

### Human Testing

All human testing was conducted with the approval of the Research Ethics Board at Sunnybrook Health Sciences Centre in Toronto, and with the free and informed consent of the volunteer participants. All participants were right-handed as assessed by the Edinburgh Handedness Inventory (Oldfield, 1971); native English speakers; free from any past or present neurological or psychiatric impairments; and recruited from the population of graduate students at the University of Toronto.

#### Desktop Tests Outside the MRI system

**Figure 2:**
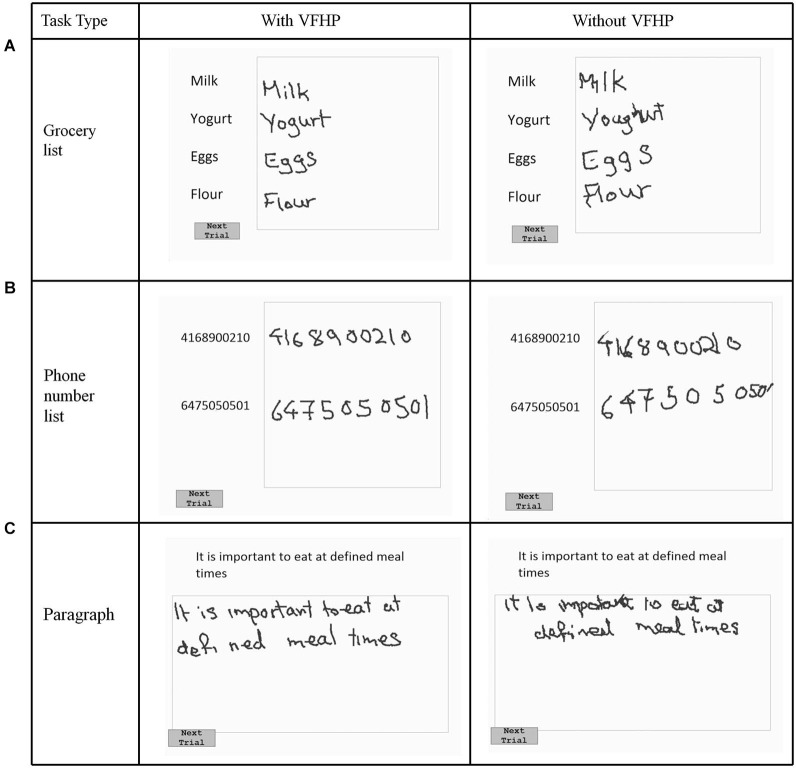
**Visual stimuli for Experiment One, involving copying (A) a grocery list; (B) a phone number list; and (C) a paragraph.** Tablet responses are shown for participant two.

**Figure 3:**
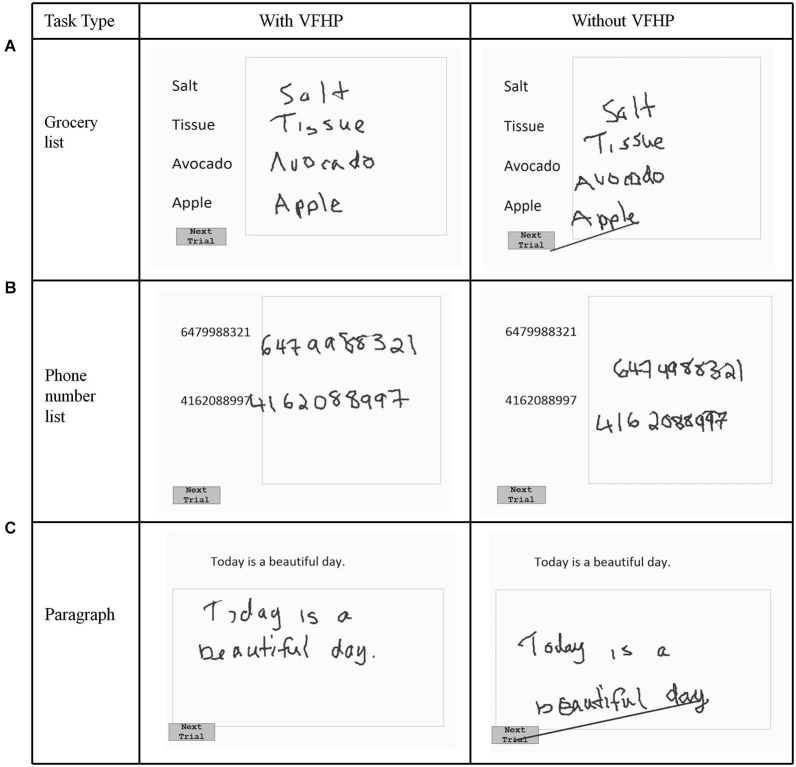
**Visual stimuli for Experiment One, involving copying (A) a grocery list; (B) a phone number list; and (C) a paragraph.** Tablet responses are shown for participant three.

Nine volunteers (age: 20–35, 4 male, 5 female) participated in the first phase of testing (experiment one), which explored the effectiveness of VFHP during complex tablet interactions in the form of handwriting tasks. Following an approach similar to that of Werner et al. (2006), who developed a series of NP tests of handwriting tasks involving a digitizing tablet, three simple writing tasks were developed that are commonly used in everyday activities (Figures 2, 3). The tasks involved (a) copying a grocery list; (b) copying phone numbers; and (c) copying a paragraph. Each task was conducted in a set of trials. For the first two tasks, participants were required to copy four grocery items and two phone numbers, respectively. Visual stimuli were listed on the left side of the display screen, with a response box located on the right side next to the list for handwriting. Participants were required to signal that they had completed each trial and were ready to move to the next by using the stylus to touch within a small box labeled “Next Trial” at the bottom left corner of the display. The procedure was similar for paragraph copying except that the box for handwriting responses was located below the paragraph. For each task, a wait interval of 5 s was included between each trial, consisting of a white screen with a central black fixation cross. Each task was repeated four times.

Subjects received training on using the tablet prior to performing handwriting tasks. This involved practicing one trial of each writing task. Each participant performed the three writing tasks in sequence using the tablet system in two different conditions: “with VFHP” and “without VFHP”. The order that these conditions were administered was randomized across the participants. The “with VFHP” condition involved use of the tablet with the revised system design described above. The “without VFHP” condition involved use of the system with the video camera disconnected, and stylus interactions based on proprioception. In both cases the participants were assessed while sitting upright at a desk where the tablet was placed on the desk surface in front of a computer screen. Participants were instructed to interact with the tablet as quickly as possible while maintaining accuracy, while viewing the computer screen and not looking at their hand. The surface of the tablet and hand/stylus interactions were also obscured from vision using an opaque cloth drape.

The outcomes of Experiment One were assessed qualitatively by visual inspection of handwriting performance, and quantitatively through three parameters, calculated from the force sensor data recorded during tablet interactions. These parameters included the total writing time; the total time the stylus was in contact with the tablet surface; and the mean stylus force applied by the subject against the tablet. The force sensor data was sampled and recorded every 25 ms during task performance using the custom program written in E-prime software. Differences in these three parameters for the “with VFHP” and “without VFHP” conditions across the cohort were then assessed statistically using the Wilcoxon signed-rank test, with a Bonferroni *post-hoc* correction for multiple comparisons.

**Figure 4:**
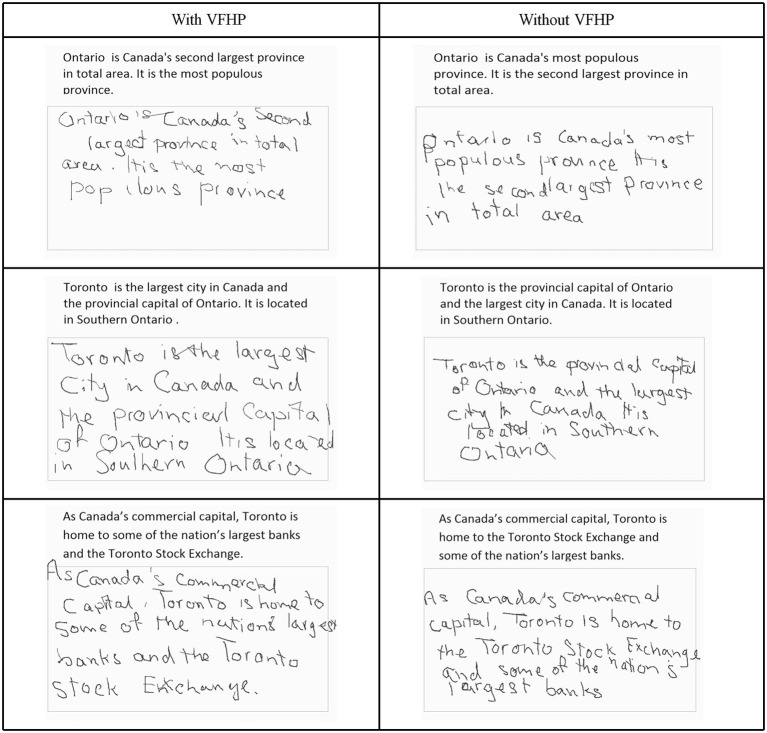
**Example visual stimuli for Experiment Two**. Tablet output is shown for participant one.

**Figure 5:**
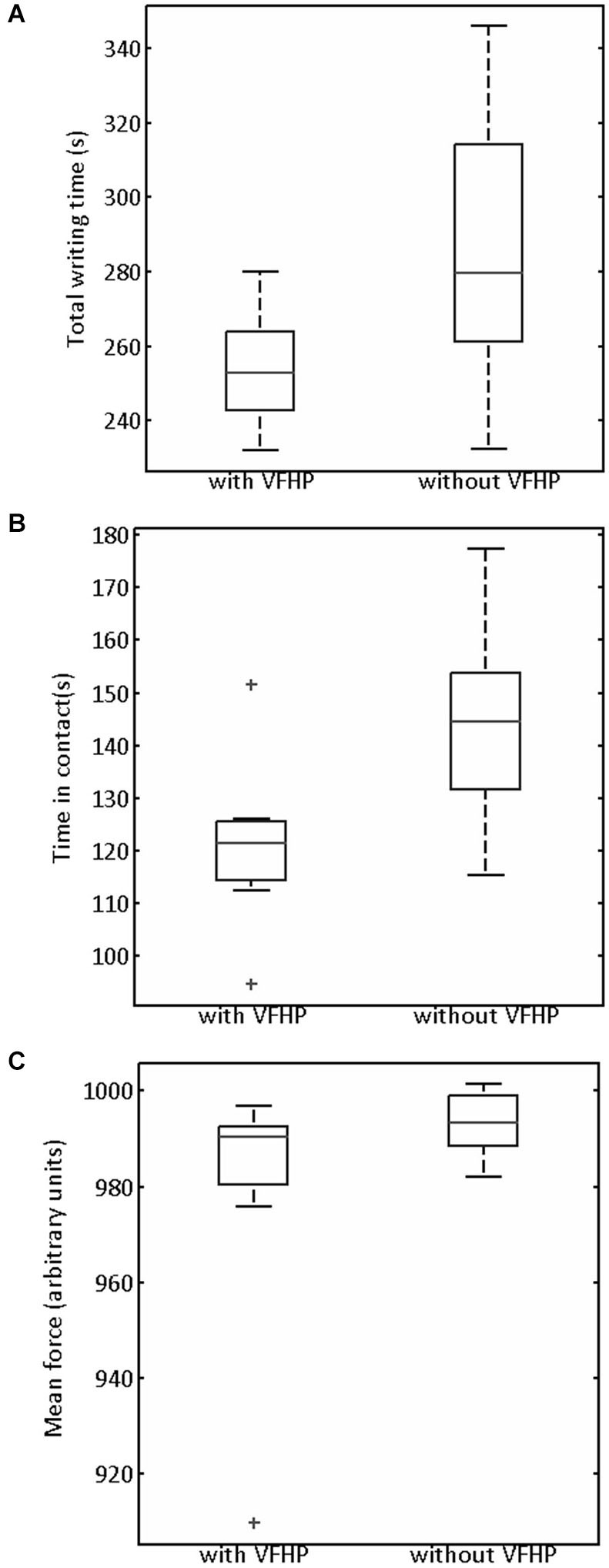
**Box and whisker plots of (A) Total writing time, (B) Time stylus was in contact with tablet surface, (C) Mean stylus contact force for participants performing all tasks in Experiment One with VFHP and without VFHP**. On each box, the center horizontal line shows the median, and the edges of the box are estimates of the 75th and 25th percentiles. The error bars extend to the most extreme data points not considered outliers (2.7 times the sample standard deviation under the assumption of a normal distribution). Outliers are shown as crosses.

Using the same set-up, an additional cohort of 9 individuals (age: 20–35, 4 male, 5 female) performed Experiment Two, which was designed to investigate learning effects for the two tablet conditions using a slightly modified version of the paragraph copying test (Figure 4). The rationale for performing this experiment was that if performance differences were observed in the prior behavioral experiments (judged as likely) then these differences could arise from participants having insufficient time to learn how to interact with the tablet in one of the two conditions. To investigate whether this was the case, it was necessary to study tablet performance over a longer time duration. Each trial of the learning task consisted of copying three paragraphs of differing content. There were four trials conducted for each of the tablet conditions. In successive trials, words within the paragraph for each trial were kept identical, but re-ordered with preservation of grammar and syntax. This procedure helped the participants to maintain attention to the task and to minimize copying by memory. Participants were instructed to lift the stylus off the tablet once they had finished copying each paragraph, such that force data could be used to index task completion. Prior to starting the experiment, each participant was provided with a brief instruction session so that they were familiarized with (but not extensively trained on) the task requirements for operating the tablet system in the “with VFHP” and “without VFHP” conditions. This was achieved by having the participant draw a circle, draw three parallel lines, and write the letter “A” in each condition. Participants then started the experiment using one tablet condition (either “with VFHP” or “without VFHP”), performed one trial of the four modified paragraph tasks, then switched to the other tablet condition for the next trial, and continued to alter between conditions until all trials were completed. The start condition was randomized across participants. After completing the writing tasks, subjects were asked to complete a questionnaire that involved rating different aspects of ease-of-use during performance in both tablet conditions. Task performance in both conditions was subsequently assessed primarily in terms of the time that the stylus remained in contact with the tablet, as this parameter provided the most distinction between the two tablet conditions in Experiment One (see Results, Figure 5). Specifically, the stylus contact time across trials for the two tablet conditions was assessed using a three-way mixed-effects Analysis of Variance (ANOVA), with tablet condition (with VFHP, without VFHP) and trial number (1–4) as fixed effects, and participants as the random effects.

#### Testing Inside the MRI System

Brain activation maps associated with writing performance in the “with VFHP” and “without VFHP” conditions were also investigated in a pilot fMRI experiment involving two healthy young adult participants (age: 20–35, 2 female) who met standard fMRI inclusion criteria (no ferromagnetic implants, no claustrophobia, etc.,). The participants operated the tablet while lying supine in the magnet bore, with the tablet mounted on a support stand that lifted the touch-sensitive surface away from the torso. The support ensured that respiratory motion did not move the tablet and thereby affect behavioral performance. The tasks were the same as those described in the Experiment One, with the exception that the fixation duration between trials, consisting of a white screen with a central black fixation cross, lasted 10 s for the fMRI experiments. The duration for each task was 35 s and participants were required to lift the stylus off the tablet once they had finished copying each task, rather than pressing a “Next Trial” button.

All imaging was conducted at 3.0 Tesla using a research-dedicated whole-body MRI system (MR750, GE Healthcare, Waukesha, WI), with a standard 8-channel head coil. An angled mirror was attached to the head coil so that the participant could view visual stimuli on a rear-projection screen mounted at the rear of the magnet bore. Anatomical MRI was undertaken using standard three dimensional (3D) fast spoiled gradient echo imaging. Anatomical data was collected with inversion time (TI) 300 ms, repetition time (TR) 7.0 ms, echo time (TE) 3.1 ms, flip angle 15°, field of view (FOV) 22 × 22 cm, matrix = 256 × 192, number of slices = 190, slice thickness = 1 mm. These images subsequently served as an anatomical underlay to the color maps of brain activity generated from fMRI data. Functional MRI was undertaken using a T2*-weighted spiral in/out pulse sequence (Glover and Law, 2001) to record brain activity via the blood oxygenation level dependent (BOLD) effect. All fMRI data were collected with repetition time (TR) 2 s, echo time (TE) 30 ms, flip angle 70°, field of view (FOV) 20 × 20 cm, matrix = 64 × 64, number of slices = 30, slice thickness = 4.5 mm. Cardiac and respiratory signals were measured during fMRI using a photoplethysmograph attached to the finger of the left hand and a respiratory belt strapped around the torso, respectively.

Statistical parametric maps (SPMs) of brain activity were calculated from the fMRI data using Analysis of Functional Neuroimaging (AFNI) freeware (Cox, 1996). Initial data pre-processing included rigid-body motion correction to register the fMRI time series data to a reference image (Cox, 1996). The reference image was taken as the 8th image in the time series of the first fMRI run. Cardiac and respiratory noise were regressed from the fMRI data using a retrospective correction algorithm (Glover et al., 2000). This step was followed by slice timing correction, consisting of an interpolation procedure that accounted for the slight differences in acquisition times between image slices. Spatial smoothing was then applied using a 6 mm full width half maximum (FWHM) Gaussian filter to increase signal-to-noise ratio (SNR) in each image (Friston et al., 1995). Brain activity was estimated using a standard general linear model (GLM) including a set of polynomial low-frequency detrending regressors, motion regressors and the task timing (Friston et al., 1994). The resulting SPMs consisted of Student t values, characterizing the brain regions with statistically significant BOLD signal change between tablet performance and rest conditions. The SPMs were thresholded using a False Discovery Rate method (Genovese et al., 2002) at *q* = 0.001. Anatomical images were aligned with a standard brain template, ICBM 452 T1, in Talairach atlas space (supplied with AFNI), and the SPMs overlaid on the anatomical images.

## Results

Figures 2, 3 show representative tablet output for Experiment One from two participants performing trials of the grocery list, phone number, and paragraph copying tasks in the “with VFHP” and “without VFHP” conditions. It is evident that handwriting is less cramped, more legible, and located better spatially within the response boxes in the “with VFHP” condition, compared to the “without VFHP” condition.

Generally the handwriting kinematics were very similar for the grocery list, phone number, and paragraph copying tasks conducted in Experiment One. Accordingly, the parameters quantifying behavioral performance were summed across the three tasks for each participant and for each tablet condition. Figure 5 shows the resulting box-and-whisker plots of total writing time, time the stylus was maintained in contact with the tablet, and mean stylus contact force for participants using the tablet to perform the three tasks with VFHP and without VFHP. Compared to performance without VFHP, performance with VFHP showed less variability and a trend toward reduced total writing time (Figure 5A) whereas statistically significant reductions were observed in total time in contact with the tablet and in mean stylus force (Figures 5B,C; *p* < 0.05, corrected).

Figure 4 shows tablet output for one participant performing the modified paragraph copying tasks of Experiment Two, using the two tablet modes. Qualitatively, handwriting showed similar characteristics as for the participants shown in Figures 2, 3, with improved placement and legibility when the tablet was used with VFHP. In addition, performance with VFHP in Figure 4 was also characterized by improved positioning of the stylus to start writing in a reasonable location within the response boxes, and better handwriting spacing. With VFHP, the participant was able to use the whole response box for paragraph copying. Without VFHP, handwriting was more closely spaced and confined away from the edges of the box.

**Figure 6:**
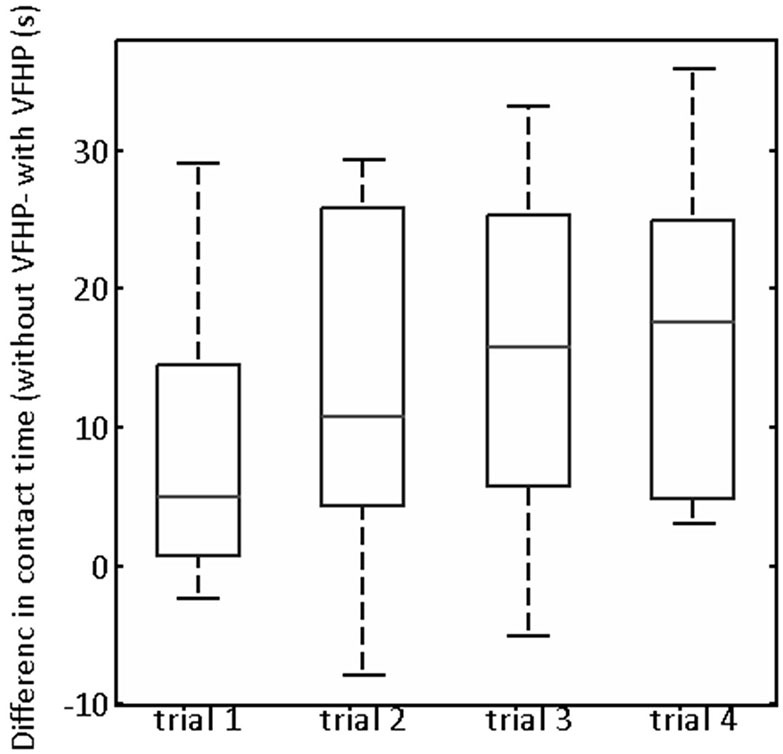
**Box and whisker showing the difference in contact time (“without VFHP” minus “with VFHP”) for participants undertaking paragraph copying in Experiment Two**. On each box, the center horizontal line shows the median, and the edges of the box are estimates of the 75th and 25th percentiles. The error bars extend to the most extreme data points not considered outliers (2.7 times the sample standard deviation under the assumption of a normal distribution). Outliers are shown as crosses.

Turning to quantitative analysis of the time that the stylus was in contact with the tablet surface in Experiment Two, the only statistically significant ANOVA result was a main effect of tablet condition (*p* < 0.05, corrected). To illustrate, Figure 6 plots the difference in stylus contact time between the two tablet conditions (“without VFHP” minus “with VFHP”) across each of the four trials. For each trial, the median difference over all participants was greater than zero, indicating that performance with VFHP required less time in which the stylus was in contact with the tablet. Although small, a trend is observable that the median difference increased and reached an approximate plateau of 15 s by the third trial. By the fourth trial, all participants performed using less stylus contact time with VFHP than without. On further inspection of the force data, it was determined that the histogram of stylus contact force values was very similar when performing with the two tablet conditions (data not shown). Consistent with Figure 6, the primary difference between the histograms was that null force values were substantially more frequent when participants performed with VFHP compared to performance without VFHP. These findings were also supplemented by participants taking significantly less time over all trials to complete Experiment Two when they performed with VFHP (Wilcoxon signed-rank test, *p* < 0.05).

**Figure 7:**
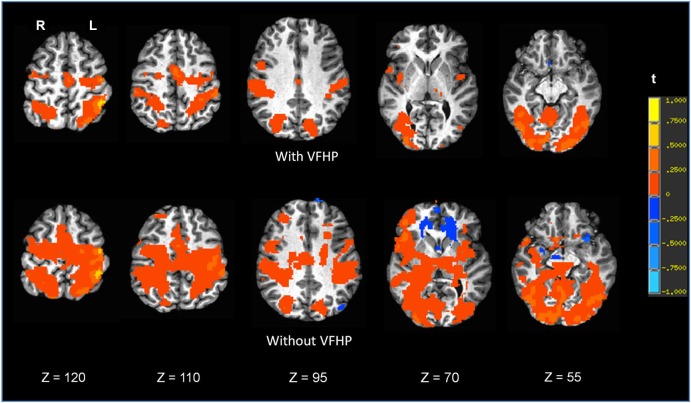
**Brain activity for a single participant copying paragraphs vs. rest, for the two different tablet conditions (“with VFHP” and “without VFHP”)**. Axial slice locations are indicated by *z* values in Talairach coordinates. R = right, L = left.

**Figure 8:**
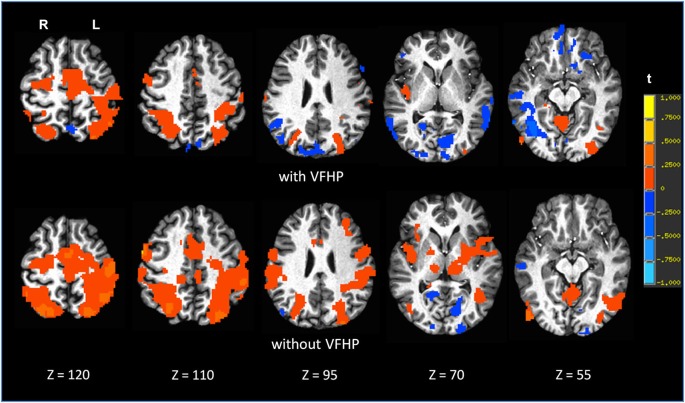
**Brain activity for a single participant copying grocery lists vs. rest, for the two different tablet conditions (“with VFHP” and “without VFHP”)**. Axial slice locations are indicated by *z* values in Talairach coordinates. R = right, L = left.

Figures 7, 8 shows brain activity in the form of SPMs for participants using the tablet with and without VFHP. Both participants performed with legible handwriting and with negligible amounts of head motion during fMRI, during the two tablet conditions. For both participants and both tablet conditions, the temporal standard deviations of the predominant components of motion “nodding” rotation and displacement in the superior to inferior direction did not exceed 0.4° and 0.4 mm, respectively, as estimated from the motion correction algorithm used in fMRI data pre-processing. Color overlays illustrate statistically significant activity that was generated while paragraphs (Figure 7) and grocery lists (Figure 8) were copied in relation to visual fixation. Similar findings were observed in both cases: when participants used the tablet with VFHP, brain activity was limited to a relatively small set of focal regions, as typified by complex sensorimotor tasks. These focal regions included areas in the left-lateralized primary somatosensory and motor cortex; as well as the bilateral supplementary motor area; bilateral parietal areas, and bilateral primary visual and visual association areas. In comparison, performance without VFHP was associated with much more extensive activity, characterized by areal expansion of the regions identified above, as well as the inclusion of additional brain regions such as the bilateral thalamus and basal ganglia, and right-lateralized prefrontal cortex.

## Discussion

The present work has described the development and validation of an fMRI-compatible tablet system that enables users to perform handwriting and drawing tasks as their time-varying brain activity is assessed, with improved interactivity in comparison to an earlier tablet design. The improved interactivity is achieved through use of the tablet in an augmented reality environment that includes VFHP, rather than the previous approach that relied heavily on proprioception. The revised design was relatively straightforward to implement in proof-of-principle from the hardware and software standpoint, enabling real-time integration of camera video, tablet responses, and computerized visual stimuli with good quality. None of the subjects reported noticed latency between their executed movements and representation in augmented reality. The current setup successfully provided VFHP in the augmented reality display for all participants, who exhibited a variety of skin tones, including one individual with dark brown skin. Although found to be unnecessary in the present work, standardized templates can be used in the future for skin tone detection for different ethnicities if the segmentation algorithm given in Equation 1 fails in a particular instance. Further technical improvements are also possible: for example, the augmented reality video data were not spatially scaled correctly in world coordinates, causing some residual spatial conflict (but not temporal conflict) between vision and proprioception; some users reported that video of the hand limited their ability to view stimuli on the display, and others reported handwriting difficulty because the tip of the stylus was rather blunt and thus difficult to localize for fine control. These issues represent only some of the opportunities for more technically-oriented research and development. From a basic neuroscience perspective, it is also worth noting that the revised tablet prototype can easily be adapted to permit novel fMRI studies of motor adaptation by appropriate manipulation of the video camera data (e.g., introducing time lag between proprioceptive inputs and VFHP, and adjusting the spatial reference frame for VFHP in relation to executed movements such as a spatial shift, magnification or reduction in the size of the hand, rotation, or mirror transformation).

Although the present work opens up many new research opportunities, the immediate outcome is that the group results of behavioral testing and fMRI examples with the current revised tablet prototype indicate important performance enhancements in comparison to the earlier design. Discussed further below, these enhancements were accompanied by anecdotal reports that using the tablet with VFHP was “less effortful” and “easier” than without VFHP.

The latter sentiments were born out by both qualitative and quantitative examination of handwriting performance in two separate experiments involving desktop behavioral tests conducted outside an MRI system. Qualitatively, handwriting performance using the tablet with VFHP was less cramped, more legible, neater, and located in space more appropriately in comparison to use without VFHP (Figures 2–4). These subjective observations were reinforced by quantitative analyses of handwriting kinematics, showing that using the tablet with VFHP was associated with reduced time in contact with the tablet, and lower time-averaged contact force with the stylus. The results are consistent with different strategies of interaction during the two tablet conditions. When interacting without VFHP, participants tended to rely on prolonged stylus contact with the tablet to help in determining where to position their responses. In particular, several participants made long lines in their responses without VFHP to facilitate pressing the “Next trial” boxes in the corner of the display (e.g., see Figure 3). In contrast, interactions with VFHP were performed by focusing on where to move the stylus tip through space sometimes in the air, such as when moving to the appropriate starting position for writing a word or number and sometimes in contact with the tablet, while making an actual stylus response. The “with VFHP” strategy was associated with better handwriting with a slight reduction in completion time (Figure 5A), although the reduction was not statistically significant in Experiment One after correction for multiple comparisons.

Experiment Two supported and extended the results of the first experiment, involved multiple trials of the modified paragraph copying task. Again, the quality of handwriting was better when participants used the tablet with VFHP in comparison to use without VFHP. The primary quantitative observation in Experiment Two was the main effect of tablet condition on stylus time in contact with the tablet surface, which was significantly longer when the tablet was used without VFHP. Differences in the rates of learning to use the tablet either with or without VFHP were found to be minor across the participants. In addition, participants took significantly less time to complete Experiment Two when using the tablet with VFHP. This result was more robust than the analogous finding in Experiment One, likely because the second experiment involved longer passages of text.

Overall, the behavioral results are consistent with participants achieving more naturalistic (ecologically valid) handwriting performance when using the revised tablet system in the VFHP condition. The results are also consistent with the findings of Ghilardi et al. (1999, 2000) and Slavin et al. (1999) who showed that vision of the hand prior to movement is required to update internal representation of the starting point in the workspace and to plan movement direction.

Experiments One and Two are further supported by fMRI results for two young healthy adult participants (Figures 7, 8). Compared to performing handwriting with VFHP, performance without VFHP showed much more extensive brain activity. This is consistent with the notion that if VFHP is not provided, placing heavy reliance on proprioception for tablet interactions is more effortful, or a more complex, challenging task that places more demands on cognitive and sensorimotor system resources to support and maintain adequate performance (Mochizuki et al., 2009). The performance gains inherent to the revised tablet system in relation to the original tablet design (Tam et al., 2011) have a number of implications. Regarding work that has been published with the original tablet system, a number of the experiments were designed such that issues of tablet interaction did not have a major influence on the interpretation of brain activity. This was achieved, for example, by studying performance on different tasks requiring tablet interaction and analyzing the resulting differences in brain activity (Callaert et al., 2011), according to the common “cognitive subtraction” paradigm (Friston et al., 1994). However, going forward, it should be recognized that the revised tablet system is a more appropriate platform for establishing the neural correlates of various NP tests that require writing and drawing, as the revised tablet elicits behavior that is more ecologically valid for typical NP tests, and is thus more usable without subtraction of a time-consuming “tablet-usage control task”. This assertion can easily be tested in future research involving fMRI studies where cohorts of participants perform established NP tests with different versions of the tablet. Such work is obviously essential, as the brain activity maps in Figures 7, 8 are primarily intended to demonstrate pilot results and feasibility. It is not unreasonable to hypothesize that such future fMRI studies will show activity that is closer to what is expected of specific NP tests on neuropsychological grounds, when the tablet is used with VFHP compared to usage without VFHP. Using the revised tablet with VFHP should also enhance activation within ventral premotor cortex and parietal cortex, areas that code the spatial location of visual stimuli within arm-centered coordinates, playing an important role in controlling and reaching movements (Graziano and Gross, 1998; Graziano, 1999; Snyder, 2000; Newport et al., 2001). Other results from the laboratory are starting to support this hypothesis, consistent with the tablet with VFHP providing more naturalistic behavioral performance, and a network of brain activity that is more reflective of real-world handwriting task performance outside the magnet (Karimpoor et al., 2014).

Lastly, there is the question of performing tablet-based fMRI studies involving patients with impaired brain function. If, as expected, it is ultimately determined in a larger cohort of healthy individuals that greater neural resources are required to support tablet interactions with the original tablet system than with the revised system including VFHP, then there is greater likelihood that patients with diffuse disease, or with lesions that impair proprioception, will have difficulty performing tablet interactions with the original system. The fMRI-compatible tablet was originally conceived as useful tool for characterizing brain activity in patients; however, potential difficulties that patients may have either in learning to use the tablet, or in making stylus interactions, will potentially confound the interpretation of activation maps. The concern is that in addition to performing the particular task of interest, they will be performing a challenging sensorimotor task at the same time. For example, in patients with traumatic brain injury or AD, decreased ability to divide attention between multiple tasks, or generalized reduction in the speed of mental processing strongly argue for making the task of interacting with the tablet as naturalistic and ecologically valid as possible.

Based on the above concerns and the positive results of the present work, we will be moving toward applying the revised tablet system with VFHP in fMRI studies of patient populations in the near future. In particular, as a natural extension of our focus on handwriting tasks, the next immediate step will be to establish the brain activity associated with the NP tests developed by Werner et al. (2006) in cohort fMRI studies of healthy young and elderly adults. These data, when combined with those of other tablet-based fMRI studies of NP tests sensitive to dementia, will provide an objective baseline of behavioral/brain function relations for ultimate comparison with fMRI studies of patients with MCI and AD, with the goal of improving characterization of disease and disease progression.

## Conflict of Interest Statement

Simon J. Graham, Tom A. Schweizer, Stephen Strother, Fred Tam, Mahta Karimpoor, 2013. Systems and methods for providing visual feedback of touch panel input during functional magnetic resonance imaging and interventional magnetic resonance imaging of the brain. Submitted to the United States Provisional Patent, patent pending.
